# The 10th anniversary of the publication of genes and environment: memoir of establishing the Japanese environmental mutagen society and a proposal for a new collaborative study on mutagenic hormesis

**DOI:** 10.1186/s41021-016-0073-5

**Published:** 2017-03-01

**Authors:** Shizuyo Sutou

**Affiliations:** Shujitsu University, 1-6-1 Nishigawara, Naka-ku Okayama-Shi, 703-8234 Japan

**Keywords:** AF-2, Collaborative Study Group of the Micronucleus Test, CSGMT, 2-(2-furyl)-3-(3-nitro-2-furyl)acrylamide, JEMS, Linear no-threshold model, LNT, Mammalian Mutagenicity Study group, MMS, Mutagen

## Abstract

The Japanese Environmental Mutagen Society (JEMS) was established in 1972 by 147 members, 11 of whom are still on the active list as of May 1, 2016. As one of them, I introduce some historic topics here. These include 1) establishment of JEMS, 2) the issue of 2-(2-furyl)-3-(3-nitro-2-furyl)acrylamide (AF-2), 3) the Mammalian Mutagenicity Study Group (MMS) and its achievements, and 4) the Collaborative Study Group of the Micronucleus Test (CSGMT) and its achievements. In addition to these historic matters, some of which are still ongoing, a new collaborative study is proposed on adaptive response or hormesis by mutagens. There is a close relationship between mutagens and carcinogens, the dose-response relationship of which has been thought to follow the linear no-threshold model (LNT). LNT was fabricated on the basis of *Drosophila* sperm experiments using high dose radiation delivered in a short period. The fallacious 60 years-old LNT is applied to cancer induction by radiation without solid data and then to cancer induction by carcinogens also without solid data. Therefore, even the smallest amount of carcinogens is postulated to be carcinogenic without thresholds now. Radiation hormesis is observed in a large variety of living organisms; radiation is beneficial at low doses, but hazardous at high doses. There is a threshold at the boundary between benefit and hazard. Hormesis denies LNT. Not a few papers report existence of chemical hormesis. If mutagens and carcinogens show hormesis, the linear dose-response relationship in mutagenesis and carcinogenesis is denied and thresholds can be introduced.

## Introduction

When the members of the board of trustees of the Japanese Environmental Mutagen Society (JEMS) were asked if they would contribute their papers to Environmental Mutagen Research (EMR), the former title of Genes and Environment (G&E), when they write papers, more than half answered no. They would rather contribute them to Mutation Research, Mutagenesis, or Environmental and Molecular Mutagenesis. The main reason was that papers in EMR were not cited in PubMed. When I was the editor-in-chief of EMR (1998–1999), I tried to make EMR a PubMed-citation journal, but my trials and efforts came to naught. EMR was a quarterly magazine and consisted of Japanese and English articles. A total annual number of original papers might not reach the criteria of citation by PubMed. EMR was changed to Genes and Environment (G&E), a fully English journal, in 2006. Minako Nagao, editor-in-chief, made a great contribution to G&E. In spite of her ardent efforts, however, citation in PubMed was not successful. The editor-in-chief was replaced by Takashi Yagi in 2011, and M. Nagao became the production editor. As she retired from this position, I took over the job from her in 2014. As the production editor, I looked through all of the accepted papers and checked them for errors and for format, so as not to deviate from the instructions to authors. One comment from me is the deletion of keywords from the title so as to avoid duplication. G&E became an open access journal in 2015, and I was freed from the position of production editor. Recently, I was delighted to learn that PubMed decided to cite G&E papers. From my viewpoint, this is a dream come true and more congratulatory than the 10th anniversary itself. As I am one of JEMS members from the start, let me make some remarks on the occasion of the PubMed citation and the 10th anniversary of G&E.

## Memoir of establishing the Japanese environmental mutagen society

JEMS was established at the National Education Center in Tokyo on August 21, 1972. The number of participants at the first meeting was 147. Active members are 11 as of May 1, 2016. They are Hikoya Hayatsu, Yasumoto Kikuchi, Taijiro Matsushima, Tohru Shibuya, Hiroyasu Shimada, Takashi Sugimura, Noriho Tanaka, Hiroshi Tanooka, Hideo Tezuka, Makoto Umeda, and I. At first, JEMS was started as the Environmental Mutagen Research Association for the first 6 years. The program and the list of participants at the first meeting were in my hands; I offered them to the JEMS’ office so as not to be lost.

The organizer-in-chief of the first JEMS meeting was Yataro Tajima, who gave an opening address at 9:30. It was followed by a congratulatory address by E.B. Freese, the then president of the American Environmental Mutagen Society. Fourteen papers were presented at the meeting. Each speaker presented his paper for 25 min. It may be of interest to learn what the top researchers at that time spoke of. Japanese titles are translated into English. Hikoyuki Yamaguchi (Tokyo University): Chromosomal aberrations by antibiotics in plants, Hidetoshi Yoshida and Yukimasa Shiraishi (National Institute of Genetics): Chromosomal aberrations by cadmium in humans, Shigeo Iwahara (National Institute of Health Sciences): Mutation induction in bacteria by food-associated substances, F.J. de Serres (National Institute of Environmental Health Sciences): Mutation induction in radiation-sensitive strains of *Neurospora crassa*, Sohei Kondo (Osaka University): Molecular mechanisms of mutations, Takeo Suzuki (Institute of Public Health): Hazardous substances in human environments, Tsuneo Kada (National Institute of Genetics): Screening methods for chemical mutagens based on the theories of chemical mutagenesis, Yasuhiko Shirasu (Institute of Environmental Toxicology): Toxicity issues of pesticides, Hideya Endo (Kyushu University): Chemical carcinogenesis and mutation, Mamoru Saito (Tokyo University): Roles of natural carcinogens in the environment, Fuminori Yanagisawa (Tokyo Medical and Dental University): Consideration of teratogenicity by alkylbenzene sulfonate, Tsuyoshi Kajiwara (Takeda Pharmaceutical Co. Ltd.): Mutagenesis testing and teratogenicity, Ujihiro Murakami (Institute for Developmental Research): relationship between teratogens, mutagens, and carcinogens, and W.W. Nichols and R.C. Miller (Institute for Medical Research, Camden): Anaphase as a cytogenetic method in mutagenicity testing.

A close relationship between mutagenicity and carcinogenicity was a major concern in these days. Carcinogenicity testing was time-consuming, labor-intensive, and costly. Researchers made efforts to develop detection methods for mutagens as substitutes for detection of carcinogens. As a result, a widely used food additive was found to be a mutagen and this created a social problem as shown below.

## 2-(2-Furyl)-3-(3-nitro-2-furyl)acrylamide (AF-2) boosted JEMS activity

Mutagenicity and carcinogenicity was the major issue of AF-2 in the 2nd JEMS meeting, organized by Yataro Tajima and held at the National Institute of Genetics in Mishima City in 1973. Seven papers out of 17 were associated with AF-2. Some insisted that AF-2 was not a carcinogen and others refuted this assertion. There was a very hot debate. AF-2 is an amorphous reddish powder. AF-2 is a stable wide-spectrum antibiotic, and bacteria do not attain tolerance or resistance to it. It is effective at very low dose levels and long-lasting. In 1965, AF-2 was substituted for nitrofurazone, a food additive approved in 1950, and used widely in foods such as hams, sausages, tofu, sweetened bean paste, and so forth. AF-2 would be an ideal food preservative if it lacked mutagenic and carcinogenic properties. The carcinogenic potential of AF-2 was tested, and negative results were obtained in Donryu rats and ICR/JCL mice [1]. AF-2 was found, however, to induce chromosomal aberrations in cultured human cells [2] and showed mutagenic and DNA-modifying effects on bacteria [3]. AF-2 spanned two epochs in the history of mutation research. First, the discovery of AF-2 mutagenicity forced a re-examination of its carcinogenicity. As a result, AF-2 was found to be carcinogenic in ddY mice [4], and AF-2 was prohibited for use as a food additive in 1974. Second, while AF-2 was mutagenic in *E. coli* B/rWP2 Trp^−^, the inability to detect AF-2 mutagenicity with *S. typhimurium* strains TA1535, TA1536, TA1537 and TA1538 [3] prompted B.N. Ames to develop the new tester strains, TA98 and TA100 [5]. The rodent micronucleus test of AF-2 reviewed by the Gene-Tox Program was inconclusive and the test was classified as “inadequate” [6]. We demonstrated that AF-2 is clastogenic in the micronucleus test using MA/Ae mice [7], though its clastogenicity was weak.

Critical remarks were that AF-2 induced forestomach cancer, but humans do not have the forestomach as rodents do, and that doses of AF-2 were so high that albino mice were colored by AF-2. The oral LD_50_ is 475 mg/kg in mice at 7-day observation. AF-2 at a concentration of 0.2% in the diet (1/3 of LD_50_) for up to 2 years was not carcinogenic [1], but carcinogenic at 0.45% (3/4 of LD_50_) for 18 months [4]. Thus, AF-2 was carcinogenic at high doses and not carcinogenic at low doses. The issue of AF-2 presents a big contemporary problem, i.e., whether or not results obtained with high doses are proportionally applicable to responses with low doses. In other words, are there thresholds in carcinogenesis? This will be discussed later.

## Establishing the mammalian mutagenicity study group and its achievements

It is not too much to say that JEMS’ activities are largely supported by those of subgroups such as the Mammalian Mutagenicity Study Group (MMS), the Bacterial Mutagenicity Study Group, and the Study Group of Mutation Mechanisms. The Environmental Epigenomics Society and the Study Group of Non-mutagenic Carcinogens have suspended their activities at the present time. Since I was involved in the establishment of MMS, let me have a say.

MMS was established in 1982 by merging the Dominant Lethal Test Seminar (organizers: Kiyoshi Tsuchikawa, Yasumoto Kikuchi, and Tohru Shibuya) and the Micronucleus Test Research Association (organizers: Motoi Ishidate, Jr. and Yasumoto Kikuchi). An ad hoc committee held a meeting in February 1982, and adopted the name of MMS. The objectives of MMS were to scrutinize in vivo mutagenesis testing systems and, by supporting development of the research area, to contribute to safety evaluation in humans. At the meeting held at the occasion of the 11th annual meeting of JEMS in October 1982, in Shuzenji, organized by Yukiaki Kuroda, action programs were adopted and sub-committees were formed: 1) to do collaborative studies, 2) to have workshops, and 3) to examine protocols. MMS decided to have biannual meetings. MMS had the 69th meeting in December 2016, while JEMS had the 45th meeting in this November 2016.

As for 1) to do collaborative studies, a spot test group and a micronucleus test (MN) group were organized at first. Collaborative studies by MMS have expanded to more than 20 studies as follows: (1) Mouse spot test, (2) Sex-related difference in MN, (3) Strain difference in MN, (3) Administration route difference in MN, (4) Treatment times in MN, (5) Peripheral blood MN using supravital staining with acridine orange, (6) MN using IARC (International Agency for Research on Cancer) carcinogen, (7) Aging and spontaneous and induced MN, (8) Rat peripheral blood MN, (9) Examination of genotoxicity using transgenic animals, (10) Development of MN using organs other than the bone marrow such as the liver, intestine, skin, and gonads, (11) Risk assessment of genotoxicity with special reference to mitomycin C, (12) Development of MN coupled with the 4-week repeat dosing test, (13) Toxicogenomics using mice and rats, (14) relationship between in vivo and in vitro genotoxicity and carcinogenicity (ongoing), (15) In vitro MN using human cells, (16) Development of MN using the liver of juvenile animals, (17) Examination of in vitro and in vivo comet assays, (18) Conduct of an international collaborative in vivo comet assay using rats, (19) Development of MN using the liver and digestive tracts of mature rats given repeated doses (ongoing), and (20) Development of Pig-assay (ongoing). The results of these collaborative studies have been reported in more than 100 papers.

**Fig. 1:**
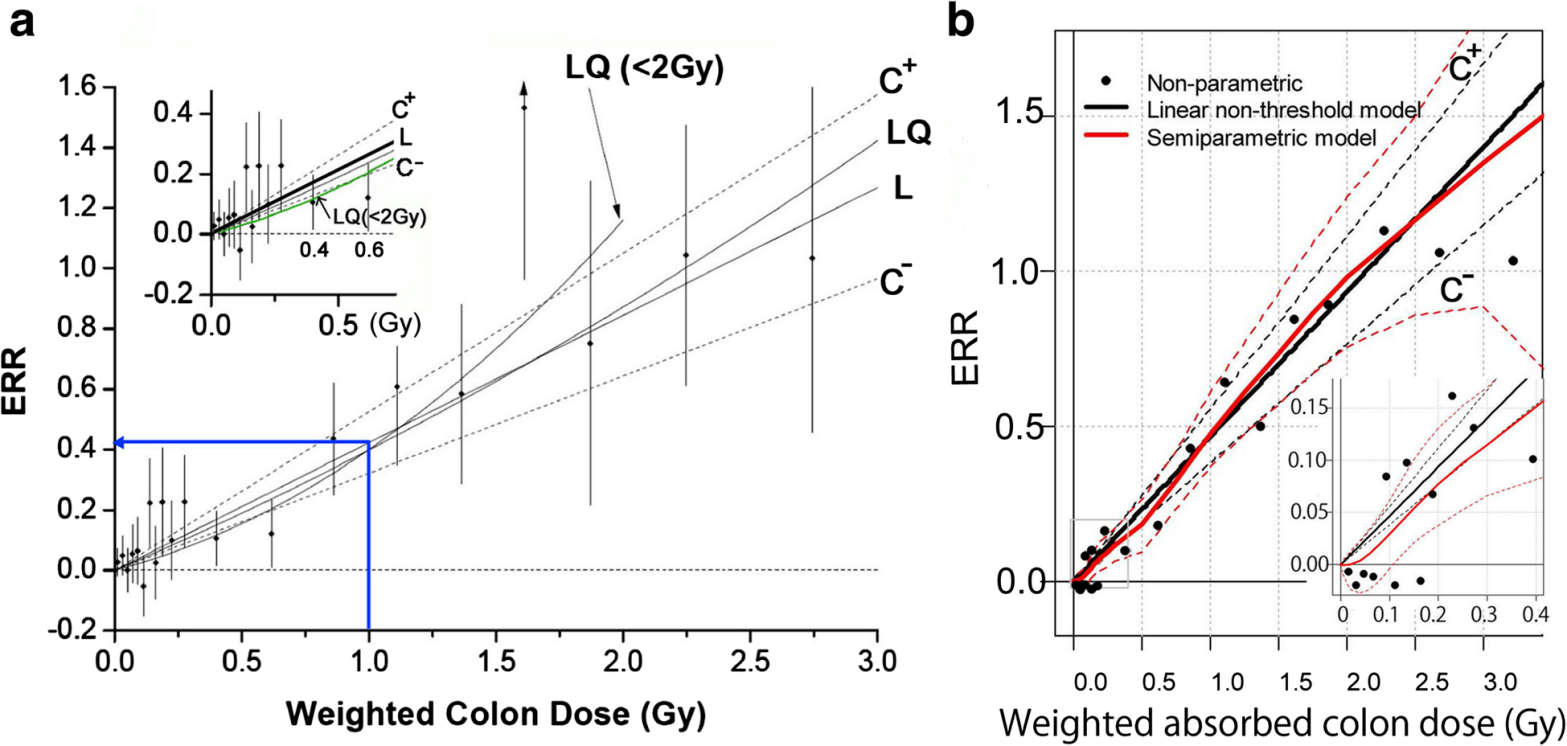
Excess relative risk (ERR) of solid cancer in atomic bomb survivors. **a** [31], L: linear fit, LQ: linear-quadratic fit, C^+^ and C^−^: 95% CI (confidence interval) to L. Assumed ERR is 0.42 at age 70 when people of age 30 were exposed to 1 Gy (**a**, *blue arrow*). Inset shows ERR at dose range 0–0.6 Gy. See that only one point is between C^+^ and C^−^, unusually low ERR at 0.4 and 0.6 Gy, and LQ (<2 Gy) comes under C^−^ (*green line*). **b** [33], comparison of conventional LNT (*black lines*) with a Bayesian semiparametric model (*red lines*). C^+^ and C^−^ are 95% confidence interval for LNT and 95% credible interval for the semiparametric model. As for the definition of non-parametric data (filled circle), see the reference [33]. Inset shows ERR at dose range 0–0.4 Gy. See that LNT has only one point between C^+^ and C^−^, while eight points (ERR at 0.6 Sv is not shown) are located between C^+^ and C^−^ in the Bayesian model, which predicts a threshold and hormesis. Significant increase of ERR is observed over 0.1 Gy. Figures are presented with permission from Radiation Research (**a**) and John Wiley and Sons (**b**)

As for 2) to have workshops, we had a workshop on how to judge and detect spots using the positive control of stuffed mice before conducting the mouse spot test. Kiyoshi Tutikawa was the main instructor. Several workshops were carried out before the conducts of MN. For example, bone marrow samples from several mice treated with mitomycin C were combined and stained by Makoto Hayashi and slides were distributed to 25 participants. Results are shown in Fig. 1 of my review paper [8]. Although some fluctuations were seen at the first trial, these workshops must have contributed to build up participants’ skills and to standardize methods of MN.

A workshop on chromosomal aberrations was also held. On the basis of this workshop, an atlas book was published [9]. Approximately 1,000 photos were supplied by Yoshiaki Kimura and Shinya Hitotsumachi (Takeda Pharmaceutical Co. Ltd.), Koji Kondo (Shionogi & Co. Ltd.), Seiji Sato and Shizuyo Sutou (NRI Life Science), Minoru Sawada and Toshio Sofuni (National Institute of Health Sciences), Hiroyasu Shimada and Chiharu Hattori (Daiichi Pharmaceutical Co., Ltd.), Noriho Tanaka and Koji Yamakage (Food and Drug Safety Center); 180 photos were selected from them. Texts of the atlas book were written by Motoi Ishidate, Jr., Toshio Sofuni, and Makoto Hayashi (National Institute of Health Sciences), and Naomichi Inui (Japan Tobacco Inc.).

MMS had its own journal, MMS Communications (MMSC) (ISSN 0918–5976). This was started as a kind of newsletter. MMSC No. 1 was prepared on the occasion of the 14th MMS meeting in November 1988. It was then published annually until 1991 (No. 4). In 1992, Nos. 5 and 6 were issued for the biannual MMS meetings. The first original paper was published by Naohiko Higashikuni and Shizuyo Sutou (ITOHAM FOODS Inc.) in No. 7 issued in 1993 [10]. The Nos. 1–7 constitute Vol. 1 of MMSC. Would-be No. 8 was issued in November 1993, but this volume was titled “Reference materials for the 24th biannual MMS meeting” because it contained original, unpublished papers that were going to be contributed to other journals. As collaborative studies went on, lots of data were accumulated and we felt a need to have a medium in which to present our own data. Biannual MMSC was opened to the public and Vol. 2, No. 1 was issued in 1994. The managing editors were Makoto Hayashi, Takeshi Morita (Nippon Glaxo, Ltd.), Shizuyo Sutou, and Hironobu Yajima (Snow Brand Milk Products Co., Ltd.). MMSC published supplemental issues twice. The first one contains details of two papers (summaries of the 6th and 7th collaborative studies) that were presented to the 6th International Conference on Environmental Mutagens (ICEM) at Melbourne, in February, 1993. This was distributed to the participants. The second one contained presentations at the Tokyo pre-meeting, held on December 7–8, 1992, for the International Workshop on Standardization of Procedures in Genetic Toxicology, the plenary session of which was held at the 6th ICEM. MMSC was published biannually until Vol. 4, 1996.

My colleagues and I published 10 papers in MMSC [10–19]. After 1996, MMSC was merged to Mutation Research. This merger left a contribution route to Mutation Research through MMS. Contributors send their manuscripts to one of MMSC editors, who ask two peer reviewers to examine each of them. Peer-reviewed manuscripts are sent to the editor-in-chief of Mutation Research. I have communicated several papers to Mutation Research through this route. Now that G&E has become a PubMed-citation journal, this route could be closed.

## Establishing the collaborative study group of the micronucleus test and its achievements

As mentioned above, a small group to examine MN protocols was established at the Shuzenji meeting in 1982. Members were Hiroyasu Shimada (chief), Makoto Hayashi, Yoshisuke Nishi (Japan Tobacco Inc.), Tohru Shibuya, Noriho Tanaka, and myself. We examined 9 guidelines including those of European Economic Community (EEC), US Environmental Protection Agency (EPA), Federal Republic of Germany (FRG), International Commission for Protection Against Environmental Mutagen and Carcinogen (ICPEMC), Japanese Ministry of Health and Welfare (JMHW), Organisation for Economic Co-operation and Development (OECD), and United Kingdom Environmental Mutagenesis Society (UKEMS). Close examination of these protocols for about 2 years revealed that these were not based on experimental data, but were deduced mainly from chromosomal aberration tests. As a result, we decided to carry out collaborative studies and to make MN protocols that are based on data.

I proposed to conduct a collaborative study to examine sex-related differences at first, because animal numbers could be halved if there were no sex-differences or if the differences were negligible. This proposal was suggested by my research into sex-determination mechanisms conducted at Susumu Ohno’s laboratory in City of Hope Research Institute in 1980–1981. The sex difference [20] was followed by strain difference [21], administration dosing difference [22], and so on. I was in charge of preparation of manuscripts for these early papers. The author was the Collaborative Study Group of the Micronucleus Test (CSGMT). The total number of papers produced by collaborative studies is more than 100, but the exact number is not in my hands.

The 5th ICEM was held at Case Western University, Cleveland, Ohio, on July 10–15, 1989. I had a chance to present our large body of MN data from CSGMT. The audience seemed to be deeply impressed by our large body of data. I think this provided a precedent for data-based protocols and for international experimental collaborations thereafter. After my presentation, Motoi Ishidate, Jr. told me that I won fame internationally. I answered, not me but CSGMT.

China had planned to hold the 6th ICEM in 1993; however, the crackdown known as the Tiananmen Square Massacre occurred on June 4, 1989. Chinese troops with rifles and tanks killed unarmed Chinese civilians in Beijing. The ICEM board decided not to have the 6th ICEM in China, saying that an international meeting could not be held in such a barbarous county. So the 6th ICEM was held in Melbourne in 1993, hosted by the Australia and New Zealand Environmental Mutagen Society (ANZEMS). The Chinese Environmental Mutagen Society hosted the International Symposium on Environmental Mutagenesis and Carcinogenesis in Shanghai in May 1991. I was an invited speaker and presented a paper, “Strain difference in the micronucleus induction among different strains of mice with a special reference to MS/Ae mice.”

F.J. de Serres, an editor of Mutation Research, asked me via Motoi Ishidate, Jr. to review the achievements of CSGMT. Earlier topics described above are shown more precisely in this review paper published in 1996 [8]. This might also be useful to learn the history of JEMS, MMS, and CSGMT in early days.

## Summary of achievements and characteristics of MMS and CSGMT

1. MMS is one of sub-groups of JEMS and consists of around 150 members. CSGMT is one of sub-groups of MMS that carries out MN. The number of members varies depending on subjects. MMS has biannual meetings and results obtained by CSGMT and other groups are reported there.
2. Workshops held before collaborative studies were useful to propagate standard techniques, which supported the acquisition of qualified data.
3. A body of qualified data contributed to the establishment of domestic and international protocols and guidelines, e.g., for the International Conference on Harmonization of Technical Requirements for Registration of Pharmaceuticals for Human Use (ICH) and OECD. Subjects associated with regulatory sciences were of great concern for industries, constituting a factor to invite participants from industries.
4. A large number of participants made it possible to collect lots of data in a limited period; the burden to each participant was not so heavy and one could take part in collaborative studies fairly easily. Collaboration among governmental, academic, and industrial researchers was readily achieved.
5. The results of collaborative studies were published in journals. The publication provided the participants with credit. I am not sure, but around 20 people might get the doctorate degree using results of collaborative studies in part.
6. The domestic activities of MMS and CSGMT were integrated, at least in part, into the international framework of guideline setting.

This section is written mainly in the past tense. But activities of MMS and CSGMT are still ongoing, and the situation after changing from the past to the present tense would mostly hold.

## Proposal of a new collaborative study: adaptive response or hormesis by chemicals

### Fabricated linear no-threshold model

Muller discovered that X-rays can induce mutations in *Drosophila melanogaster* [23]. Atomic bombs were dropped on Hiroshima and Nagasaki in 1945. The consequent fear of nuclear warfare might have supported the award of a Nobel Prize to him in 1946 because he believed that even the smallest amount of radiation is hazardous to human genes. Muller knew of the existence of a threshold, but he asserted that there is no threshold dose in his Nobel Prize lecture [24]. He defended his faked linear no-threshold (LNT) model with the prestige of the Nobel Prize to the bitter end.

Standard Oil Co. Inc. was established by John Rockefeller in 1870. The Rockefeller Foundation (RF) was threatened by the discovery of atomic energy. In 1954, RF chose to finance six projects to evaluate atomic radiation. RF asked the U.S. National Academy of Sciences (NAS) to organize the whole program, which was conducted under the auspices of Bronk, president of the Rockefeller University, president of NAS, and an RF trustee. The Genetics Panel (GP) was established in NAS in 1954 and was chaired by Weaver, an RF officer. GP consisted of 17 members, 13 of whom were geneticists including Muller. Most members believed that all doses of radiation were harmful, irreversible, cumulative, and linearly acting, no significant discussion occurred [25]. GP recommended LNT on June 12, 1956 [26], abandoning the threshold of 500 mGy/y since 1934. The next day, the New York Times, owned by an RF trustee, reported on LNT on the front page. Other media followed. Soon after its publication, several leading biologists asked GP to provide documentation to support the LNT. GP informed the president of NAS, Bronk, that it would not provide any documentation; right from the start, they did not have relevant data.

### Conversion of genetic risk to cancer risk and from radiation to chemicals

X-rays induced mutations at high doses in the *Drosophila* sex linked recessive lethal test and LNT was fabricated on the data observed in insect sperm that lack repair systems. Actually, responses to X-rays in *Drosophila* were not linear and showed thresholds and hormesis [27]. Lewis concluded that radiation induces leukemia using Atomic bomb survivors’ data [28]. The National Council of Radiation Protection and Measurement (NCRPM) proposed the use of LNT for cancer risk assessment in 1958. In 1958, the U.S. Food and Drug Administration (FDA) introduced the Delaney Clause, which allows no carcinogens in foods. This ideal, zero-risk rule was soon confronted with reality. Scientific advancement demonstrated that there is no absolutely safe food in the world; the unrealistic Delaney Clause was abolished in 1996.

The Environmental Protection Agency of the U.S.A. (EPA) is involved in the regulation of carcinogens under several laws. As risk cannot be eliminated completely, EPA introduced a concept of balance between risks and benefits [29]. For risk assessment of a suspect carcinogen, experimental data using animals are important. The data are estimated by assuming LNT, i.e., linear no-threshold dose-response relationship. In spite of accumulated scientific knowledge, LNT has not been revised and a safe level of exposure has not been set for chemical carcinogens still now.

### Lifespan study of atomic bomb survivors does not support LNT

The most important data to support LNT are data of Lifespan Study of atomic bomb survivors (LSS). The Biological Effect of Ionizing Radiations (BEIR) of NAS asserted that the dose-response relation in cancer risk was linear and supported LNT [30]. The latest published result of LSS [31] insists that the dose-response relation is linear and there are no thresholds (Fig. 1, a). Both LNT and LSS, however, harbor intrinsic defects.

1. Exposure doses are largely underestimated because the doses were estimated only for initial radiation, within 1 min after the atomic bomb blast. The residual radiation was neglected. It was twice as high as the initial radiation and was carried to the ground by black rain. Its effects must have lasted for days or weeks. This means that cancer risk in the LSS is largely overestimated.
2. “In-the-city-control” people, who entered Hiroshima or Nagasaki after the atomic bomb blast and were used as the negative control, were exposed to residual radiation and would not be appropriate as the negative control. Indeed, their cancer mortality rate was less than that of people in the villages northwest of Hiroshima because the “in-the-city-control” people showed hormetic effects [32].
3. Both reports say that the linear-quadratic fit is better than the linear fit (compare L and LQ in Fig. 1, a). There is no statistical significance between the two, so they insist that the dose-response is linear.
4. The BEIR and LSS reports depict dose range between 0–2 Sv and 0–3 Sv dose ranges (Fig. 1, a), respectively. The responses at higher doses, in which the dose-response curve shows a downturn, are omitted. The downturn directly negates the linear dose-response.
5. Doses <100 mSv are most important for our daily life. There are no statistical significant differences between the control and the atomic bomb survivors at these doses. The BEIR report combined all data points <100 mSv, to which more than 80% survivors belong, together into one point. This dishonest statistical trick was successful in giving the impression that the dose-response is linear and no thresholds exist. The LSS report shows all data points. Alas, 12 out of 13 data points <0.6 Sv are located outside the 95% confidence interval (Fig. 1, a), suggesting that there is no linearity in a low dose range.
6. Both the BEIR and LSS reports are based on conventional parametric analyses. Some drawbacks harbored in these analyses can be cleared when a Bayesian semiparametric analysis is used. This new analysis [33] shows that the dose-response curve is rather S-shaped or sigmoidal than linear (Fig. 1, b, red line) and there is a threshold (Fig. 1, b, inset). Eight data points out of 13 are located inside the 95% credible interval (Fig. 1, b, inset), while only one point is inside the 95% confidence interval by the conventional analyses (Fig. 1, a, inset).
7. Both leukemia [34] and solid cancers [32] incidences in the LSS are hormetic.
8. The atomic bomb survivors were exposed with high doses and high dose-rate radiation, the effects of which were critically hazardous as compare with those of low doses and low dose-rate radiation as in the case of Fukushima.

Thus, the LSS does not support LNT. Gene mutations, induced by high-dose radiation in *Drosophila* sperm, led fallaciously to LNT without relevant data. The failed germ cell mutation hypothesis was applied to somatic cell mutations by radiation without relevant data. LNT is now fundamental basis for radiation-regulatory guidelines. Then, LNT was applied to chemical carcinogenesis without relevant data. Thus, the smallest amount of carcinogens is proportionally carcinogenic on the basis of unproven inference. The linear dose-response relationship of chemical carcinogens without thresholds must be reevaluated.

### Proposal of a new collaborative study: adaptive response or hormesis by chemicals

Recently, I commented on hormesis [35]. Adaptive response, or more properly hormesis, is seen universally in many organisms including atomic bomb survivors [32, 34]. Living organisms have established efficient defense mechanisms against radiation through the evolutionary history of billions of years. Hormesis resembles the immune responses. Vaccination with attenuated viruses beforehand provides us with tolerance to virulent viruses afterwards. Hormesis can be seen in the following situations.

1. Radiation or chemicals are beneficial at low doses, but hazardous at high doses.
2. Exposure to low doses beforehand gives enhanced cell repair after exposure to high doses.
3. Exposure to X-rays gives tolerance to γ-rays. Exposure to substance A beforehand gives tolerance to substance B afterwards.

Hormesis must be applied to chemicals. Mutagens and carcinogens would not follow the manner that LNT predicts. Many papers indicate the existence of chemical hormesis. I proposed a new collaborative study on hormesis in mutagenesis at the 68th MMS meeting on June 17, 2016. Presently, a test chemical that is found to be mutagenic in one of the mutagenicity testing methods, it is branded as a mutagen. My expectation is that it might be mutagenic at higher doses, but would be antimutagenic at lower doses. In between higher and lower doses, there must be thresholds. My proposal is to examine the situation experimentally. When mutagenic hormesis is proven, a mutagen will be classified as a hormetic mutagen, indicating that the mutagen is not always hazardous. Many drug candidates might have been dropped off as mutagens during developmental process, but hormetic mutagens could be developed as drugs with less fear about mutation or cancer induction. Changes of guidelines for registration of drugs, cosmetics, pesticides, and so on are expected in the future if hormetic mutagenesis is proven.

## Abbreviations

AF-2: 2-(2-Furyl)-3-(3-nitro-2-furyl)acrylamide
BEIR: Biological Effect of Ionizing Radiations
CSGMT: Collaborative Study Group of the Micronucleus Test
EMR: Environmental Mutagen Research
EPA: Environmental Protection Agency
FDA: Food and Drug Administration
G&E: Genes and Environment
ICEM: International Conference on Environmental Mutagens
JEMS: Japanese Environmental Mutagen Society
LNT: Linear no-threshold model
LSS: Lifespan Study of atomic bomb Survivors
MMS: Mammalian Mutagenicity Study group
MMSC: MMS Communications
MN: Micronucleus test
NAS: National Academy of Sciences
